# Local opposite orientation preferences in V1: fMRI sensitivity to fine-grained pattern information

**DOI:** 10.1038/s41598-017-07036-8

**Published:** 2017-08-02

**Authors:** Arjen Alink, Alexander Walther, Alexandra Krugliak, Nikolaus Kriegeskorte

**Affiliations:** 1 MRC Cognition and Brain Sciences Unit, Cambridge, UK; 2 Department of Systems Neuroscience, University Medical Centre Hamburg-Eppendorf, Hamburg, Germany; 3 University of Birmingham, Birmingham, UK

## Abstract

The orientation of a visual grating can be decoded from human primary visual cortex (V1) using functional magnetic resonance imaging (fMRI) at conventional resolutions (2–3 mm voxel width, 3T scanner). It is unclear to what extent this information originates from different spatial scales of neuronal selectivity, ranging from orientation columns to global areal maps. According to the global-areal-map account, fMRI orientation decoding relies exclusively on fMRI voxels in V1 exhibiting a radial or vertical preference. Here we show, by contrast, that 2-mm isotropic voxels in a small patch of V1 within a quarterfield representation exhibit reliable opposite selectivities. Sets of voxels with opposite selectivities are locally intermingled and each set can support orientation decoding. This indicates that global areal maps cannot fully account for orientation information in fMRI and demonstrates that fMRI also reflects fine-grained patterns of neuronal selectivity.

## Introduction

Visual orientation is known to be represented in columnar preference patterns in the primary visual cortex (V1) at a sub-millimetre scale^1^. Kamitani and Tong^2^ demonstrated that fMRI patterns measured in V1 at standard resolution (3-mm isotropic voxels) provide information about the orientation of visual gratings. This study had a big impact in part because it suggested a sensitivity of standard-resolution fMRI to columnar-scale neuronal selectivity patterns. However, it has been proposed that V1 orientation decoding might rely on coarse-scale organizations instead^3^. In particular, several studies demonstrated slight preferences for radial orientations^4–7^, which might explain orientation decoding results. A left-tilted diagonal grating, for example, will have approximately radial orientation in the upper left and lower right quadrants, driving the corresponding quarterfield representations of V1 more strongly than the other two quarterfield representations^4^. It has been argued that this effect is necessary for fMRI orientation decoding^6^.

One way to minimize a contribution to orientation decoding from the radial-preference map is to use logarithmic spiral stimuli. A logarithmic spiral has a constant orientation relative to the radial direction, e.g. 45°. Two spirals with orientations 45° and −45°, respectively, relative to the radius are orthogonal to each other everywhere. They are also balanced about the radial direction everywhere, and thus radial preference cannot account for their decodability. However, such spirals have been shown to be robustly decodable^5, 7, 8^. In addition to radial-preference, however, there is evidence that V1 patches also respond preferentially to vertical orientation^5, 7, 8^. This global vertical preference predicts distinct global-areal patterns to be elicited by opposite-sense spirals and, thus, spiral decoding as well might be explained by global-areal-scale pattern information.

**Figure 1:**
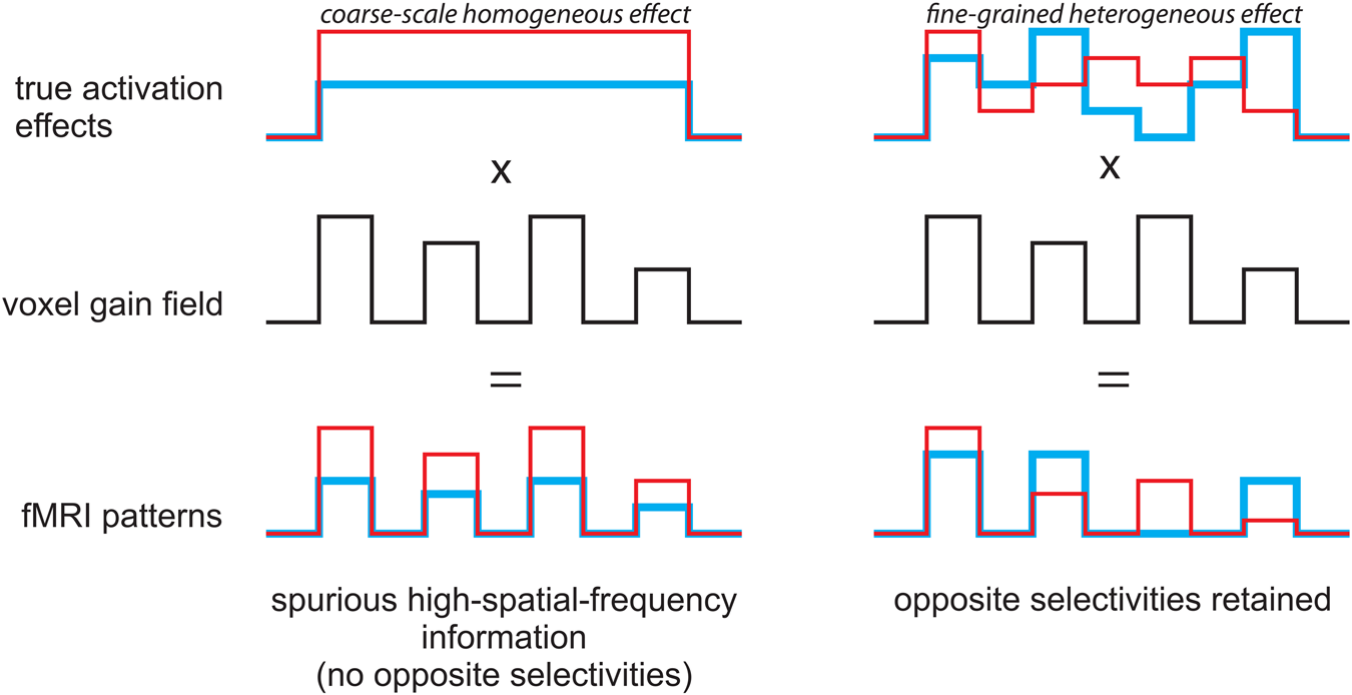
Coarse-scale neural effects can give rise to spurious high-spatial frequency fMRI pattern information in the presence of a high-spatial frequence gain field across voxels. An illustration of how differences in sensitivity to local activation across voxels (the voxel gain field) can lead to spurious high-spatial- frequency information in fMRI patterns. The left column shows the effect of a gain field on a coarse scale homogenous effect and the right column shows the effect of gain field on a fine-grained heterogeneous effect. An important property of the gain field effect is that the signs of the true activation effects are preserved. Spatial filtering analyses will suggest high-spatial frequency information in either scenario (left and right). However, the signature of a fine-grained heterogeneous effect (right) is the presence of local opposite selectivities (right only). Note that in actual data the sign of effects can be inverted by fMRI noise; this effect is not illustrated in this figure.

The aim of the current study is to test if fMRI response patterns with a grain finer than these two coarse-scale preference maps contribute to orientation decoding. The observation that orientation decodability is robust to high-pass filtering of fMRI patterns has been considered as evidence for a fine-grained contribution to fMRI orientation decoding^7, 9, 10^. Filtering analysis, however, is not able to conclusively determine whether fine-grained activation patterns contribute to orientation decoding because coarse-scale neural effects can give rise to spurious high-spatial frequency fMRI pattern information if adjacent voxels have different sensitivity to local neural activity. This effect is illustrated in Fig. 1. Differences in sensitivity (the voxel gain field) can result, for example, from partial volume sampling, with some voxels sampling mainly gray matter and others mainly white matter. A voxel gain field is not expected to invert the sign of a contrast between two stimuli. Therefore, if orientation decoding of gratings and spirals originated solely from coarse-scale radial and vertical preferences, respectively, then one would not expect voxels in a local cluster to exhibit reliable opposite preferences. Under the global areal account of grating decoding (i.e. radial preference), a small patch of V1 representing a region within one visual quarterfield should not contain voxels preferring tangential over radial stimuli. Similarly, under the global areal account of spiral decoding (i.e. vertical preference), a small patch of V1 representing a region within one visual quarterfield should not contain voxels preferring horizontal over vertical stimuli. Here we show that local voxel clusters in V1 do exhibit reliable preferences for both radial and tangential orientations (in the gratings scenario) and for both vertical and horizontal orientations (in the spirals scenario). The opposite preferences are intermingled within small patches of V1, forming a fine-grained pattern. Gratings can robustly be decoded using either only the radial-preferring or only the tangential-preferring voxels. Similarly, spirals can be decoded using either only the vertical-preferring or only the horizontal-preferring voxels. These results clearly demonstrate the reliable presence of voxels of opposite selectivity within local small patches of V1. Fine-grained fMRI patterns, thus, contribute to orientation decoding.

## Results

### Voxels with reliable opposite orientation preferences intermingle within small V1 patches

**Figure 2:**
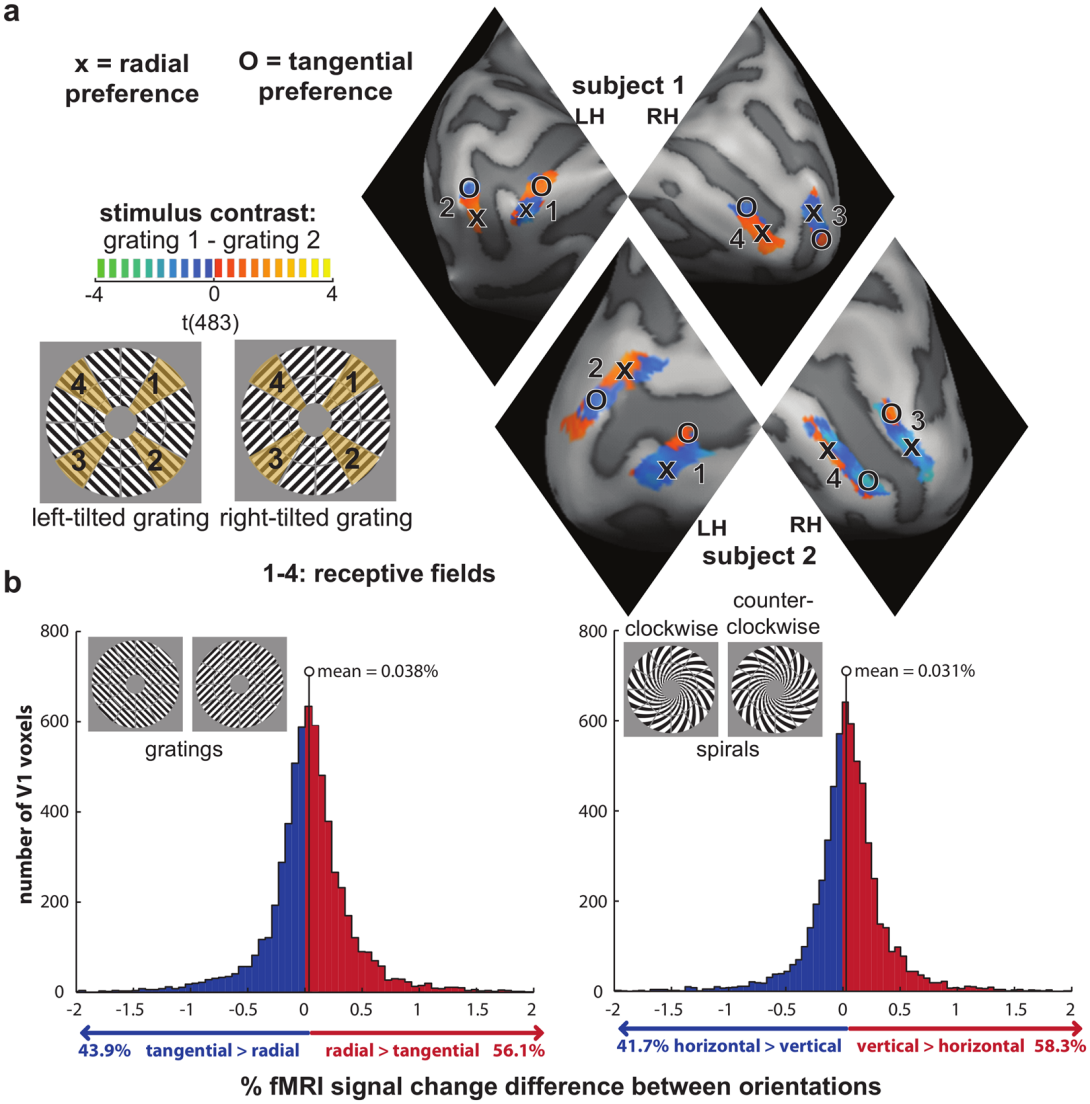
Opposite orientation preferences intermingle within quarterfield patches in V1. (**a**) A visualization of how fMRI voxels were labeled as preferring radial and tangential orientation. The contrast t maps indicate the activation difference between the two displayed visual gratings. Activation is only shown for the four within-quarterfield ROIs, which are labeled clockwise from 1 to 4. Positive and negative t-values indicate either a radial or a tangential preference depending on the visual field they are in. This we have clarified by labeling local activation clusters with 0 and X when they have a tangential and radial preference respectively. Note that the activation map is unthresholded and that no inferences are made based on it. (**b**) Histograms showing the distribution of V1 voxels that prefer radial vs tangential orientation (left) and vertical vs horizontal orientation (right). These plots are based on all voxels in all four quarterfield ROIs across all participants. (**c**) A visualization of the proportion of V1 voxels preferring radial orientation (left, red), tangential orientation (left, blue), vertical orientation (right, red) and horizontal orientation (right, blue) across subjects and quarterfield ROIs.

In order to find out if V1 contains voxels with opposite orientation preferences, we considered the responses to left- and right-tilted gratings in V1 patches at the center of the visual quarterfields (representing polar angles 45°, 135°, 225° and 315° clockwise from vertical; Fig. 2a, left). For a given patch of voxels, each grating had either a radial or a tangential orientation. Based on the patch-relative orientation of the grating stimuli, voxels were labeled as preferring radial or tangential orientation depending on which grating evoked the greater response (as visualized in Fig. 2a, right). We applied the same rationale to determine whether V1 voxels prefer vertical or horizontal orientation based on responses to the spiral stimuli. Importantly, orientation preferences were determined using training data only for both the decoding and preference-replicability analyses.

### Grating and spiral orientation can be robustly decoded

**Figure 3:**
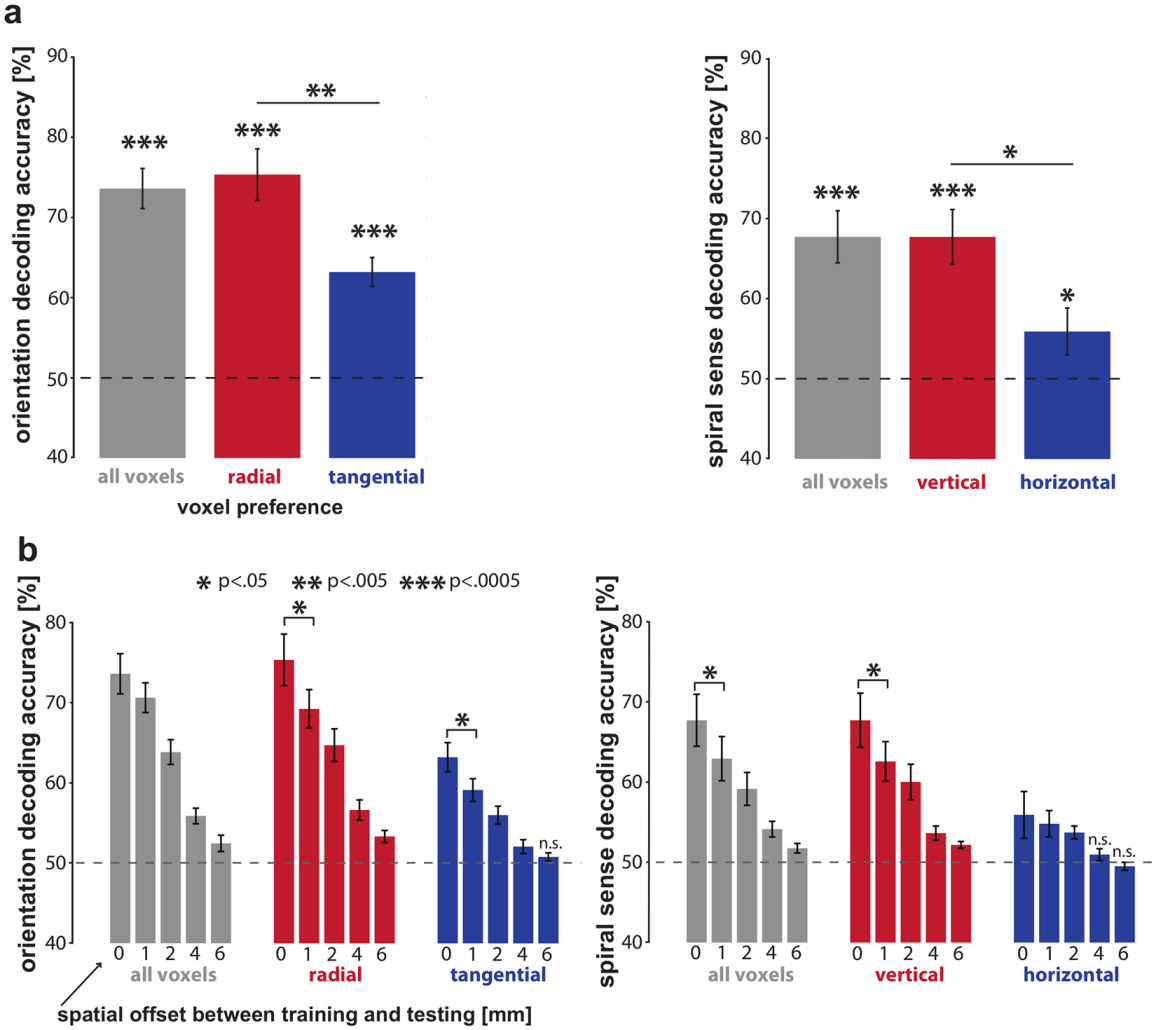
Tangential and horizontal orientation preferences on their own allow for robust orientation decoding. (**a**) Bar plots summarizing grating orientation (left) and spiral sense (right) decodability when selecting all voxels (gray bars), voxel preferring radial/vertical preference (red bars) and voxel preferring tangential/horizontal preference (blue bars). (**b**) Bar plots summarizing how grating orientation (left) and spiral sense (right) decodability is affected by spatially shifting test patterns by 1, 2, 4 and 6 mm.

First we assessed stimulus decodability using all voxels within the four patches, regardless of their orientation preference. Consistent with previous studies, decoding analyses using linear support vector machines (Fig. 3a) revealed that the two gratings are robustly decodable (74% accuracy, p < 0.0005). The two spirals, similarly, were robustly decodable (68% accuracy, p < 0.0005; Fig. 3a). For the gratings, either radial- or tangential-preferring voxels, or both sets might contribute to orientation decodability. For the spirals, similarly, either vertical- or horizontal-preferring voxels, or both sets might contribute. Note that vertical preferences cannot contribute to grating decoding, because the two gratings were balanced about the vertical orientation. Similarly, radial preferences cannot contribute to spiral decoding, because the two spirals were balanced about the radial orientation.

### Both tangential- and radial-preferring voxels support orientation decoding

Single voxel responses are noisy. Even if orientation information resulted only from a coarse-scale map of radial preference, we would expect some inverted preference estimates (apparent tangential-preferring voxels), due to the noise in the data. In order to assess whether the tangential preferences were real, we tested their reliability in the decoding framework (Fig. 3a). Importantly, orientation preference of voxels was determined independently from the test data. Spurious orientation preferences would not replicate in the test data. We found that grating orientation can be robustly decoded based only on voxels with a tangential preference (63% accuracy, p < 0.0005, one-sided Wilcoxon signed-rank test across subjects). Consistent with the previously reported slight bias in favor of radial preferences^4, 6, 7^ decoding was also possible using only radial-preferring voxels (75% accuracy, p < 0.0005) and the accuracy was significantly greater for the radial-preferring voxel set than for the tangential-preferring voxel set (p < 0.006, two-sided Wilcoxon signed-rank test across subjects). These results show that the two opposite-preference sets of voxels, which are intermingled within the patches of V1, each carry significant orientation information.

### Both horizontal- and vertical-preferring voxels support orientation decoding

We performed analogous analyses on the response patterns elicited by the spirals (Fig. 3a). We found that spiral orientation can be robustly decoded based only on voxels with a horizontal preference (56% accuracy, p < 0.04, one-sided Wilcoxon signed-rank test across subjects). Again, consistent with the previously reported slight bias in favour of vertical preferences^5, 7, 8^, decoding was also possible using only vertical-preferring voxels (68% accuracy, p < 0.0005) and the accuracy was significantly greater for the vertical-preferring voxel set than for the horizontal-preferring voxel set (p < 0.02, two-sided Wilcoxon signed-rank test across subjects). For the spirals, as well, results show that the two opposite-preference sets of voxels, which are intermingled within small patches of V1, each carry significant orientation information.

### Tangential, radial, vertical, and horizontal preferences are replicable – and opposite orientation preferences intermingled within quarterfield and eccentricity patches

**Figure 4:**
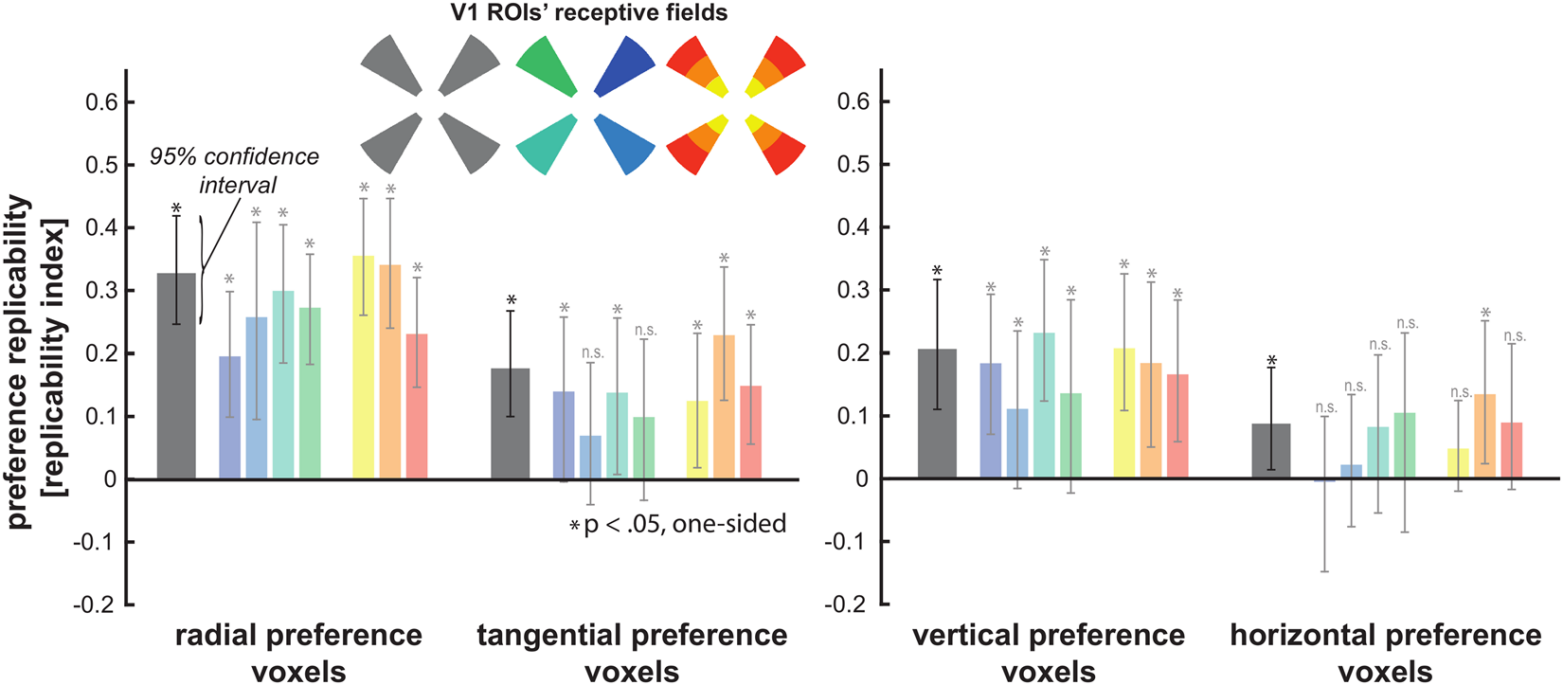
Tangential and horizontal orientation preference strength across V1 voxels replicates from training to test data. (**a**) Bar plots summarizing the average correlation between orientation preference strength across V1 voxels between training and testing data - using leave-one-subrun out cross-validation. Preference replicability is shown for all quarterfield ROIs combined (grey bars) for radial, tangential, vertical and horizontal orientation (left to right). In addition, preference replicability is shown separately for the four quarterfield ROIs (blue-green bars) and separately for the three eccentricity ROIs (yellow-red bars). Error bars depict the 95% confidence intervals based on bootstrap resampling (10.000) of the participant set.

We tested the robustness of each orientation preference more directly by determining whether orientation preference of V1 voxels in training data generalizes to test data. Results from this analysis (Fig. 4) indicate that each orientation preference is replicable when combining all four quarterfield ROIs (radial: average replicability index = 0.33, p < 0.001, Tangential: average replicability index = 0.18, p < 0.001, Vertical: average replicability index = 0.21, p < 0.001, Horizontal: average replicability index = 0.09, p < 0.02, p-values are based on bootstrap resampling (10.000) of the participant set using a one-sided test). When considering each quarterfield ROI separately, orientation replicability is significant (p < 0.05) for ten of the sixteen comparisons (Fig. 4). When considering each eccentricity-specific ROI separately, orientation replicability is significant (p < 0.05) for ten of the twelve comparisons (Fig. 4). We tested if ROI quarterfield and eccentricity had a significant effect on orientation preference replicability for each orientation in eight separate one-way ANOVAs. The outcome of this analysis did not imply a relation between ROI selection and orientation preference replicability as p-values for all eight ANOVAs exceeded p = 0.12. Results for V2 were are qualitatively similar to these V1 results (see supplementary material).

### V1 voxels exhibit subtle radial and vertical preferences

In order to assess radial and vertical preferences on a group level we estimated the response amplitude difference (in % signal change) between radial and tangential for all voxels across all participants within the four quarterfield ROIs. We plotted the histogram of the radial-tangential response difference across V1 voxels (Fig. 2b – left side; pooled across quarterfield patches and the 18 participants). The histogram shows that voxels in V1 are slightly more likely to prefer radial orientations over tangential orientations (56.1% vs 43.9%). These proportions were significantly different (p < 0.0005, two-sided across-subject t-test, test statistic: within-subject %-point difference, subject as random effect). The mean response difference between radial and tangential orientations was 0.038%-signal-change.

We performed analogous analyses for the spiral stimuli (Fig. 2b – right side), which drive each patch with either a vertical or a horizontal orientation. We plotted the histogram of the vertical-horizontal response difference across V1 voxels (Fig. 3b; pooled across quarterfield patches and the 18 participants). The histogram shows that voxels in V1 were slightly more likely to prefer vertical orientations over horizontal orientations (58.3% vs 41.7%). These proportions were significantly different (p < 0.0005, same test as above). The mean response difference between radial and tangential orientations was 0.031%-signal-change.

Both the radial-over-tangential and the vertical-over-horizontal preference effect sizes were small, less than 2% of the average fMRI response in these V1 patches for gratings and spirals, which were 2.03%-signal-change and 2.17%-signal-change, respectively. These results are consistent with the previous analysis of this data set in Alink *et al*.^7^.

### Shifting test patterns by half a voxel or more reduces orientation decodability

Freeman and colleagues^8^ recently found that the decodability of grating orientation and spiral sense is not affected by shifting activation patterns by half a voxel (1 mm) between training and testing by shifting the EPI image coordinates during data acquisition between odd- and even-numbered runs. This was taken as evidence for fMRI orientation decoding relying mainly on coarse-scale orientation preference maps rather than intermingled orientation preferences. To relate this finding to our data, we have assessed decoding performance after shifting test patterns by 1, 2, 4 and 6 mm relative to the patterns used for training. Note that in our study we shifted activation patterns after data-acquisition based on the reconstructed fMRI images - which introduces additional smoothing of the patterns. Despite this fundamental difference, this shifting method is also expected to reduce biases introduced by the exact placement of each voxel.

Our results show strong effects of spatial shifts on decoding performance (Fig. 3b). Even the minimal shift of 1 mm significantly reduced decoding performance for four out of the six voxel selections (Fig. 3b, p < 0.05). Orientation decodability for all selections was found to approach chance level for 6 mm shifts. These results are consistent with the presence of information across multiple spatial scales, including fine-grained and coarse-scale patterns.

## Discussion

The aim of the current study was to find out whether fine-grained neural activation patterns contribute to fMRI orientation decoding in the context of acquisition with a typical 3T scanner at a spatial resolution of 2 mm isotropic. Alternatively, fMRI orientation decoding might rely solely on global-areal patterns resulting from radial and vertical preferences. Previous studies used spatial-frequency filtering techniques to address this question. However, spatial-frequency filtering can be confounded by a high-spatial-frequency voxel gain field and can suggest the presence of fine-grained pattern information where there is none (Fig. 1, left). Here we exploited the fact that the voxel gain field is not expected to invert the selectivity of a voxel. Therefore, reliable opposite orientation selectivities within a small cluster of voxels indicate fine-grained pattern information (Fig. 1, right).

We investigated whether there are two separate sets of voxels in a small patch of V1 that have opposite orientation preference. To ascertain that each set has a reliable preference (and is not just inverted by noise), we performed orientation decoding on each set separately. This analysis revealed that grating and spiral orientation can be decoded based on voxel populations with either orientation preference. In addition, we demonstrate that orientation preference strength in each set of V1 voxels is replicable and that this replicability does not depend on quarterfield or visual eccentricity of V1 patches. Lastly, our results suggest that voxel sets with opposite replicable orientation preference intermingle within local V1 patches. These findings taken together, indicate that global-areal patterns evoked by vertical and radial preference in V1 are not the only source of visual-orientation information in fMRI at 3T and support the idea that fine-grained activation patterns contribute to orientation decoding.

Our results are also consistent with the previously demonstrated enhanced V1 responses to radial^4–8^ and vertical orientation^5, 7, 8^ as voxels preferring vertical and radial orientation were slightly more common than those preferring tangential and horizontal orientation. As expected, we also found that decoding performance was greater when selecting voxels preferring radial and vertical orientation for gratings and spirals, respectively. Our results, thus, replicate the presence of global-areal preferences and demonstrate that fine-grained patterns as well contribute to orientation decoding.

However, the radial bias of V1 voxels observed in this study appears to be modest when comparing it to the results reported by Freeman and colleagues^6^. This difference might be due to the fact that we collected comparably less data from eighteen participants while Freeman and colleagues^6^ collected more data for fewer participants (four, including three of the authors). This could have led to less statistical power on an individual participant level which might have biased our estimate of the proportion of radial vs tangential preferring voxels towards 0.5. In contrast to the study of Freeman and colleagues^6^, however, our results allow us to make inferences about the population from which the participants were drawn because all of our statistical tests were performed using single effect estimates per participant (random effect analysis)^11^. This is especially relevant given that the study by Maloney and Clifford^12^ indicates that a radial bias in V1 is not consistently present across participants. Another important difference between the current study and the study by Freeman and colleagues^6^ is that we used a blocked design for retinotopic mapping and the assessment of orientation preference instead of the fast temporal-encoding paradigm employed by Freeman and colleagues^6^. This might also explain the difference in the robustness of the radial preference because the fast temporal-encoding paradigm has recently been shown to artificially enhance the effect of radial bias in V1^13^.

Recently, it has been suggested that that orientation decoding might results from differences in contrast along the edges of annular gratings with different orientations^14^. Contrast along the annular edge of a grating varies as a function of the orthogonality between the grating orientation and local edge orientations. As a consequence, vertical annular gratings give rise to higher contrast at the top and bottom edge while horizontal gratings lead to greater contrast at the lateral edges. This edge effect is thought to give rise to global-areal activation differences similar to those resulting from a radial preference. However, the edge effect does not offer a simple account of spiral decoding, where edge-orientation-contrast patterns are expected to be matched between stimuli^15, 16^. Therefore, edge effects might contribute a global-areal component to grating decoding, but not to spiral decoding. Moreover, they do not account for the local intermingling of opposite selectivities.

Our finding that fMRI orientation decoding is supported by fMRI voxels with opposite orientation preferences does not imply that these orientation preferences have a salt-and-pepper spatial distribution in V1, or that standard-resolution fMRI can directly measure subvoxel columnar activation patterns^3, 17, 18^. Instead, we argue that the fine-grained orientation preferences we report might result from orientation-specific responses of veins on the scale of the fMRI voxels – similar to the finding of ocular dominance preferences in macroscopic blood vessels at 7T^10^. Veins might exhibit such a preference because their branches happen to non-uniformly sample columns preferring different orientations. Alternatively, it has been suggested that the vasculature might align itself to the functional architecture of the cortex during development with veins specifically draining from columns of a particular orientation preference^19^.

Note that our results do not invalidate the point raised by Op de Beeck^3^ and colleagues that one should be conservative in interpreting positive results from multi-voxel analyses in terms of sub-voxel sensitivity. Our results, however, do suggest that fine-grained patterns contribute to fMRI orientation decoding because effects other than a single global areal map effect give rise to orientation decodability.

In summary, we demonstrate that voxels with various preferences intermingle within small voxel clusters. In addition to global-areal patterns resulting from radial and vertical preferences and edge effects, thus, fine-grained patterns do contribute to fMRI orientation decoding at conventional resolution at 3T.

## Materials and Methods

### Stimuli and design

#### Common features of all stimuli

All stimulus types were presented within an annulus (inner radius = 1.5°, outer radius = 7.04°) centered on fixation on a mid-gray background. The annulus was divided into 36 log-polar tiles defined by twelve radial lines emanating from the center at 30° offsets and two concentric divisions exponentially spaced between the inner and outer radii (radii including inner and outer: 1.50°, 2.51°, 4.20°, 7.04°). This log-polar tiling was apparent in the form of mid-gray “grout lines” present in all stimuli. The edges between the grout lines and the grating stimuli where softened using a linear fade-out regions which covered a 0.0175 polar angles for the radial grout lines. The concentric grout lines had linear fade-out regions that scaled with eccentricity and covered 0.026, 0.044, 0.073 and 0.123° respectively for from the inner to the outer grout lines. For each stimulus type there were two exemplars, which had 90° orientation disparity at every location within the annulus. The oriented edges of all stimuli had 100% contrast. The phases of the oriented edges were randomized across presentations of the same exemplar. Note that results reported here are based on a dataset used previously^7^. In this study, however, we do not analyze fMRI responses evoked by patch-swapped grating stimuli and patch-swapped spiral stimuli (see ref. 7 for more details).

#### Gratings

The orientation of the square wave gratings was 45° clockwise and 45° anti-clockwise from the vertical. The gratings had a spatial frequency of 1.25 cycles per visual degree. This spatial frequency drives V1 strongly^20^ and ensures that even the smallest tiles of the log-polar array contains more than a full spatial cycle.

#### Spirals

We used logarithmic spirals whose edges were at a constant angle of +/−45° relative to the radius emanating from fixation. The spiral stimuli had 22 rectangular contrast cycles along the perimeter. This number of cycles along the perimeter was chosen so as to approximately match the spirals’ average spatial frequency across radii to that of the uniform gratings. The two spiral exemplars differed in sense: clockwise or anti-clockwise, lending them 90° orientation disparity at every location. Spiral stimuli are radially balanced because clockwise and anti-clockwise spiral stimuli deviate equally (45°), though in opposite directions, from local radial orientations.

#### Experimental design

Stimuli were presented to each subject in a single fMRI session comprising eight scanner runs, each of which lasted eight minutes. During each run, we presented both exemplars of one stimulus type (e.g. clockwise and anti-clockwise spirals). Subjects were presented with two runs for each stimulus type. Each run was divided into four equal subruns. Each subrun contained six stimulus blocks (three blocks for each exemplar, with exemplars alternating across blocks and the leading exemplar alternating across subruns). Each block lasted 14 s and contained phase-randomized versions of a single exemplar. During a stimulus block, 28 phase-randomized versions of the exemplar were presented at a frequency of 2 Hz. The stimulus duration was 250 ms, followed by an interstimulus interval (ISI) of 250 ms, during which only the fixation dot and a tiny task-related ring around it was visible (see Task, below).

#### Retinotopic mapping stimuli

In order to define regions of interest (ROIs) within V1, we presented dynamic grating stimuli designed to optimally drive early visual cortex. Like the main-experimental stimuli, these stimuli were based on a log-polar array (Fig. 2), but without the grout lines and with 20 patches per ring. Each patch contained rectangular gratings with a spatial period of one third of the patch’s radial width. Grating orientation and phase was assigned randomly to each patch. Over time, the phase of the gratings increased continuously (1 cycle per second) resulting in continuous motion in each patch (in different directions). In addition, the orientation of the grating increased in steps of π/6, once each second, resulting in motion direction changes within patches over time. We used five such stimuli, driving different parts of the retinotopic representations in V1: (1) a horizontal double-wedge stimulus, spanning a polar-angle range of +/−15° around the horizontal meridian, (2) a vertical double-wedge stimulus of the same kind, (3) a stimulus that covered the region driven by the main-experimental stimulus (1.50°–7.04° eccentricity), (4) a 0.5°-wide ring peripherally surrounding the main-experimental stimulus annulus (7.04°–7.54° eccentricity), and (5) a 0.5°-wide ring inside the annulus (1.00°–1.50° eccentricity). Stimuli were presented in 6-s blocks. This block length was chosen to balance temporal concentration (which increases design efficiency for long blocks due to hemodynamic buildup) and stimulus adaptation (which reduces design efficiency for long blocks due to reduced neuronal responses). The five dynamic stimuli and 6-s fixation periods were all presented 20 times each in a random sequence over a single run lasting 12 min.

### Subjects and task

#### Subjects

Eighteen healthy volunteers (13 female, age range 20–39) with normal or corrected-to-normal vision took part in this fMRI experiment. All participants gave their informed consent after being introduced to the experimental procedure in accordance with the Declaration of Helsinki. The experimental procedure has been approved by the Cambridge Psychology Research Ethics Committee (ethics reference number: CPREC 2010.52)”.

#### Task – fMRI

During all runs, including retinotopic mapping, subjects were instructed to continuously fixate a central dot (diameter: 0.06° visual angle). Centered on the fixation dot, there was a small black ring (diameter: 0.20°, line width: 0.03°), which had a tiny gap (0.03°) either on the left or right side. The gap switched sides at random moments in time at an average rate of once per 3 s (with a minimum inter-switch time of 1 s). The task of the subject was to continuously report the side of the gap by keeping the left button pressed with the right index finger whenever the gap was on the left side, and by keeping the right button pressed with the right middle finger whenever the gap was on the right side. The task served to enforce fixation and to draw attention away from the stimuli. Note that this task possibly reduces the extent in which orientation is encoded by fMRI responses in early visual areas^21–23^. We nonetheless choose this task to ensure that our experimental paradigm is comparable to those used in by the most relevant previous fMRI studies on the same topic^2, 5, 6, 8^.

### MRI measurements and analysis

#### MRI measurements

Functional and anatomical MRI data were acquired with a 3T Siemens Tim-Trio MRI scanner using a 32-channel head coil. During each main run, we acquired 252 volumes containing 31 slices covering the occipital lobe as well as inferior parietal, inferior frontal, and superior temporal regions for each subject using an EPI sequence (TR = 2000 ms, TE = 30 ms, flip angle = 77°, voxel size: 2.0 mm isotropic, field of view: 205 mm; interleaved acquisition, GRAPPA acceleration factor: 2). The same EPI sequence was employed for retinotopic mapping, during which we acquired 360 volumes. For each participant we also obtained a high-resolution (1 mm isotropic) T1-weighted anatomical image using a Siemens MPRAGE sequence.

#### Data preprocessing

Functional and anatomical MRI data were preprocessed using the Brainvoyager QX software package (Brain Innovation, v2.4). The first two EPI images for each run were discarded (affected by T1 saturation effects). After preprocessing (slice-scan-time correction, 3D head-motion correction, linear-trend removal and temporal high-pass filtering removing frequencies below 2 cycles per run), functional data for all subjects were aligned with the individual high-resolution anatomical image and transformed into Talairach space^24^ as a step toward cortex-based analysis in BrainVoyager. After automatic correction for spatial inhomogeneities of the anatomical image, we created an inflated cortex reconstruction for each subject. All ROIs were defined in each individual subject’s cortex reconstruction and projected back into voxel space. Note that we did not use Talairach space or a cortex-based common space for ROI definition and within-ROI patterns were analyzed separately in each subject.

#### Retinotopic mapping and region of interest definition

A general linear model (GLM) was fitted to the retinotopic mapping data, with five predictors for the five dynamic grating stimuli based on convolving boxcar functions with the hemodynamic response function as described by Boynton and colleagues^17^. Activation t-maps for each stimulus type were projected onto polygon-mesh reconstructions of individual subjects’ cortices. We determined the borders between V1-2 based on cortical t-maps for responses to vertical and horizontal double-wedge stimuli. Regions of interest (ROIs) were only created in the portion of V1 that was more active when presenting the dynamic grating stimulus covering the main-experimental annulus as compared to central and peripheral stimulation. ROIs were defined as patches covering the central third portion of each quarterfield’s polar range as visualized in Fig. 2. We excluded the remnant of the quarterfield area to reduce spillover of signals between V1 quarterfield representations. In addition we partitioned each of these four quarterfield ROIs into three ROIs (approximately equally sized on the cortical surface reconstruction) that represent different eccentricities (see Fig. 4 for an illustration).

#### Pattern-classifier analysis and orientation preference definition

Preprocessed functional fMRI data for the main experiment and individual ROI coordinates were imported into Matlab using the NeuroElf Toolbox v0.9c (developed by Jochen Weber, Columbia University). With this toolbox, we computed a GLM for each run of each subject, using one predictor for each stimulus type for each subrun. We also included six predictors specifying 3D head motion. Each run’s GLM, thus, yielded four t-value activity patterns for each exemplar (one per subrun). Both runs combined yielded eight t-value patterns for each exemplar, which was the input for our classifier analysis. We decoded the exemplar (two orientation variants) for each stimulus type with a linear support vector machine (SVM, using the libSVM library)^25^ using leave-two-subrun-out cross-validation^26^. Cross-validation consisted of four folds over which the first, second, third and fourth subrun of both runs were selected as independent test data. We classified stimulus type using all voxels within the quarterfield patch ROIs (gray bars Fig. 3a), using only voxels with a radial or vertical preference (red bars Fig. 3a) and using only voxels with a tangential or horizontal preference (blue bars Fig. 3a). Note that we computed voxel orientation preference based only on the training data during each cross-validation fold. Spurious orientation preferences (resulting from noise) will not replicate in the test data and therefore cannot contribute to significant orientation decoding. Voxel orientation preference was determined for each quarterfield patch by computing the mean difference of t-values between orientations (e.g. radial minus tangential) across the training subruns taking into consideration the patch’s receptive-field location. For example, a voxel in a patch representing the right upper visual quarterfield was considered to have a radial preference if t-values were greater for the right tilted than the left-tilted grating (see Fig. 2 for a visualization). For spirals, a right-upper-field voxel would be considered to have a vertical preference if t-values were greater for the counter-clockwise than for the clockwise spiral.

#### Assessing the replicability of orientation preference strength

We also tested the robustness of each orientation preference more directly by computing a contrast t-map for each stimulus contrast (grating 1 minus grating 2, clockwise minus anti-clockwise spiral) separately for training and test data. For this analysis we used the same leave-one-subrun out cross-validation approach as for the pattern-classifier analysis (see above) and we also used the same approach for labeling V1 voxels as preferring radial versus tangential and vertical versus horizontal orientation. We tested if orientation preferences replicate by determining whether orientation preference across V1 voxels (quantified by the contrast t-value) replicates for each voxel set (radial, tangential, vertical and horizontal) from training to test data. To illustrate, this analysis determined whether voxels showing a tangential preference in the training data also exhibit a tangential preference in the test data. For each participant, a preference replicability index for a set of voxels (e.g. training-set tangential-preference voxels) was computed as the inner product of the t-value vectors (one t value per voxel) between training and test data, normalized by dividing by the norms of the two vectors. Like the Pearson correlation, this index ranges from −1 to 1, but unlike the Pearson correlation it fixes the regression intercept at the origin. This requires the t values to match in sign, providing a measure of preference replicability. The Pearson correlation, by contrast, could in principle be positive even if the signs of the t values did not replicate. The index is positive to the extent that t values in the test data tend to have the same sign (and thus the same preference) and magnitude as in the training data. The index is unbiased and symmetrically distributed about 0 under the null hypothesis of no replicability. The index is expected to be negative if voxels tended to revert to the opposite selectivity (e.g. if voxels appearing to have a tangential preference in the training data exhibited a radial preference in the test data). This replicability index was averaged across the four cross-validation folds for each participant. We then tested whether replicability was positive (one-sided test modelling subject as a random effect). Statistical significance was assessed by bootstrap resampling (10,000 times) of the participant set. Preference replicability was tested using all four quarterfield ROIs combined and separately for each quarterfield ROI and eccentricity-specific ROI (see Fig. 4 for an illustration).

#### Assessment of the effect of spatial pattern shifts on orientation decodability

Testing data was spatially shifted by 0.5, 1, 2 or 3 voxels – corresponding to 1, 2, 4 and 6 mm – using shifted ROI coordinates for each patch when computing test patterns. The shift of 0.5 voxel (1 mm) was realized by spatial interpolation (average of two adjacent voxels). Data was shifted in all six directions (ventral, superior, left, right, anterior, and posterior). During this analysis classification performance was computed as the average SVM decoding accuracy across all shift-directions within each participant.

### Ethical approval and informed consent

All participants gave their informed consent after being introduced to the experimental procedure in accordance with the Declaration of Helsinki. The experimental procedure has been approved by the Cambridge Psychology Research Ethics Committee (ethics reference number: CPREC 2010.52).

## Electronic supplementary material

Supplementary Information
