# Supplementary material for: Local opposite orientation preferences in V1: fMRI sensitivity to fine-grained pattern information
Source: Sci Rep. 2017 Aug 2;7:7128. doi: 10.1038/s41598-017-07036-8 (PMC5540976; doi:10.1038/s41598-017-07036-8)

Local opposite orientation preferences in V1: fMRI sensitivity to fine-grained pattern information

Corresponding author: Arjen Alink, a.alink@uke.de

Arjen Alink, Alexander Walther, Alexandra Krugliak & Nikolaus Kriegeskorte

Supplementary Material

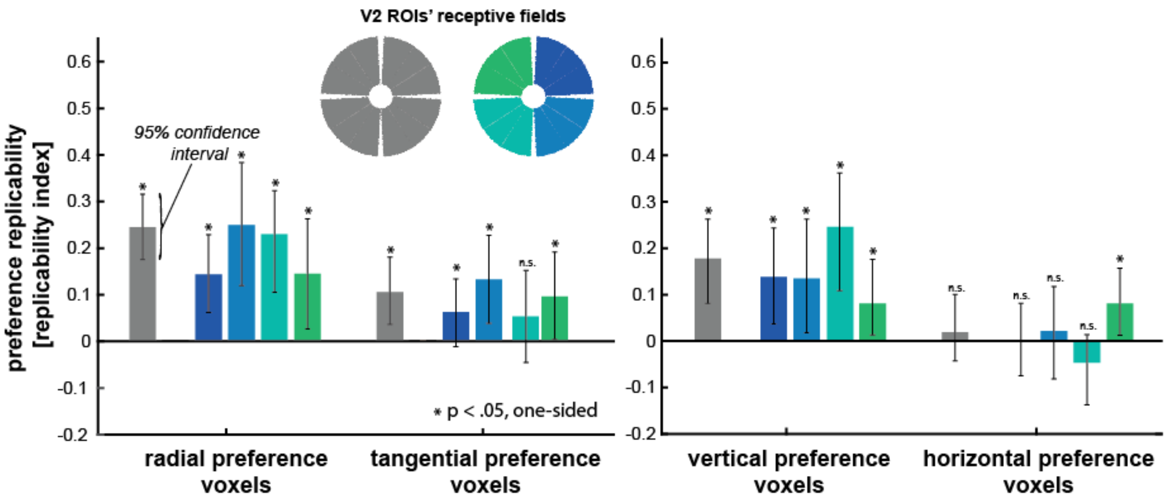

Supplemental Figure 1 Bar plots summarizing the average correlation between orientation preference strength across V1 voxels between training and testing data - using leave-one-subrun out cross-validation. Preference replicability is shown for all quarterfield ROIs combined (grey bars) for radial, tangential, vertical and horizontal orientation (left to right). In addition, preference replicability is shown separately for the four quarterfield ROIs (blue-green bars). Note that we used the entire quarterfield representation of V2 for this analysis. Errorbars depict the 95% confidence intervals based on bootstrap resampling (10.000) of the participant set.

Orientation preference replicability in V2. In V2 each orientation preference is replicable when combining all four quarterfield ROIs (radial: average replicability index = .25, $p < .001$, Tangential: average replicability index = .11, $p < .005$, Vertical: average replicability index = .18, $p < .001$, p-values are based on bootstrap resampling (10.000) of the participant set using a one-sided test) except the horizontal preference (average replicability index = .02, $p = .31$). When considering each quarterfield ROI separately, orientation replicability is significant ($p < .05$) for twelve of the sixteen comparisons (Supplemental Figure 1). We tested if ROI quarterfield had a significant effect on orientation preference replicability for each orientation in four separate one-way ANOVAs. The outcome of this analysis did not imply a relation between ROI selection and orientation preference replicability as p-values for all four ANOVAs exceeded $p=.22$.

Stimuli in their original resolution

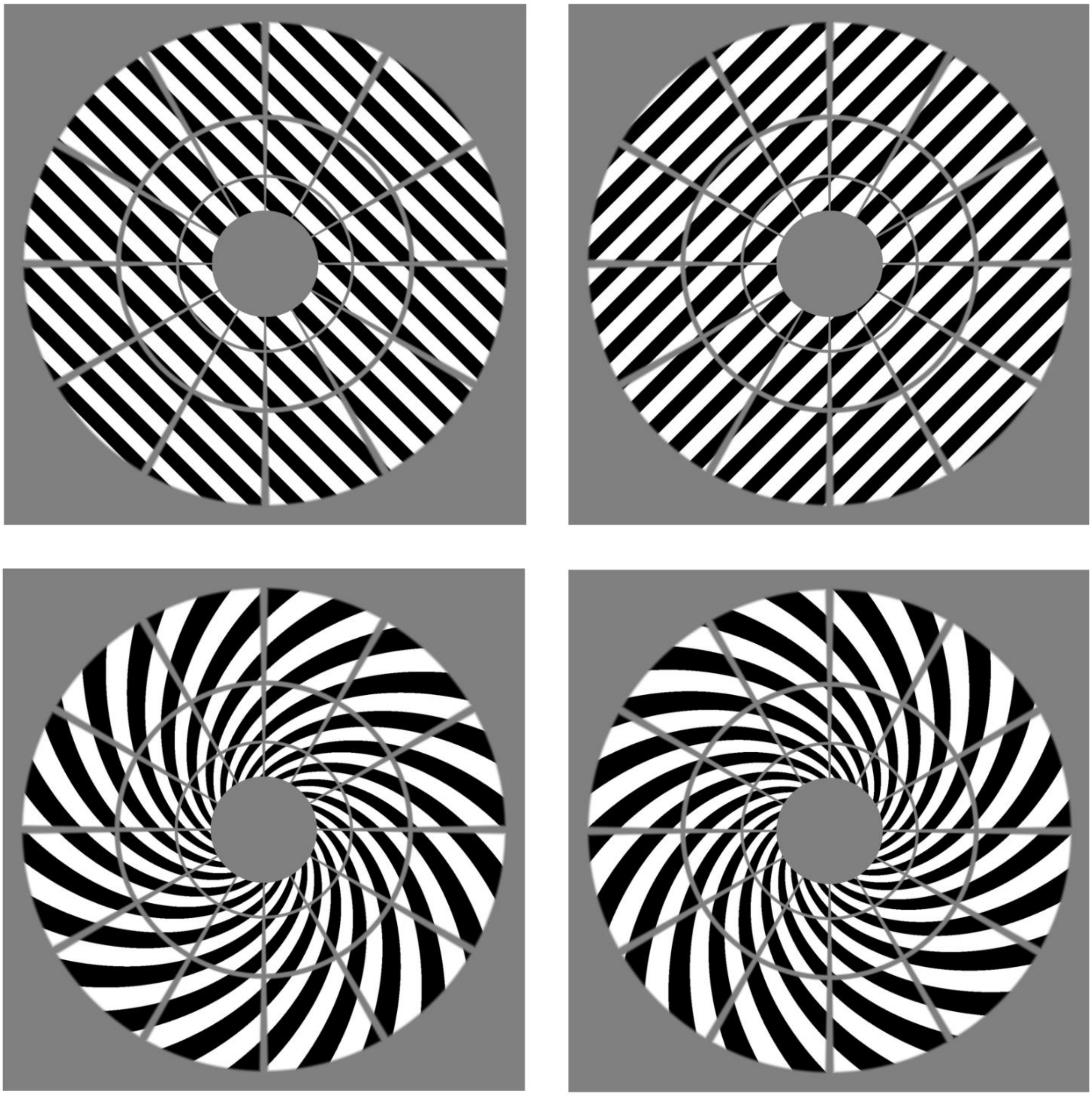

Supplement: Supplementary file 1 — Supplementary Information [file 41598_2017_7036_MOESM1_ESM.pdf]
